# Diagnosis and Management of Metastatic Breast Cancer in a 33-year-old Pregnant Female: A Case Report

**DOI:** 10.7759/cureus.5240

**Published:** 2019-07-25

**Authors:** Audrey Albach, Linden B Dixon, Sarfaraz Sadruddin, Sandra S Hatch, Quan D Nguyen

**Affiliations:** 1 Radiology, University of Texas Medical Branch, Galveston, USA; 2 Radiation Oncology, The University of Texas MD Anderson Cancer Center, Galveston, USA

**Keywords:** breast, breast cancer, pregnancy, breast imaging, oncology, radiology, radiation oncology, obstetrics, pregnancy complications

## Abstract

Despite the rarity, breast cancer diagnosed during pregnancy implies multiple therapeutic dilemmas. The initial diagnostic process can be complicated by the physiological changes that occur in the breast during pregnancy, which can further lead to a delayed diagnosis. Moreover, treatment methods, as well as treatment onset and time of pregnancy termination, remain controversial. This case report highlights some of the inherent difficulties associated with breast cancer diagnosis and treatment in a pregnant patient. It also discusses how to optimize a multidisciplinary approach to improve health outcomes for both the mother and the infant.

## Introduction

Breast cancer is the most common cancer diagnosed in pregnancy and occurs in approximately one in 3,000 pregnant women [1]. The treatment of breast cancer diagnosed during pregnancy is a challenging situation for the patient, family, and healthcare providers. The simultaneous diagnosis of breast cancer in a pregnant female adds complexity to cancer treatment recommendations and proves a significant risk to fetal development. As more women chose to postpone childbearing until middle age, the incidence of breast cancer in pregnancy is increasing and delays in diagnosis and treatment are more common. Therefore, pregnant women with breast cancer are more likely to have larger tumors, positive nodes, metastases, and vascular invasion [2]. The average patient is between 32 and 38 years of age at presentation [1].

The available data on pregnant patients with breast cancer are primarily from case reports or case-control studies due to the ethical restrictions in conducting randomized clinical trials in such patients. The aim of this case report was to evaluate the management of a patient with advanced breast cancer during pregnancy and to determine whether this patient was treated according to the appropriate guidelines, which serve to maximize both fetal and maternal outcomes.

## Case presentation

A 33-year-old female presented with an extensive history of a rapidly enlarging right breast mass. Two years ago, the patient initially presented to an outside emergency department with a six-month history of a right breast mass. The patient reported the mass around the nine o’clock position of her right breast during a breast self-examination. Other contributory symptoms included fatigue, right-sided paraspinal pain, and pleuritic chest pain. Three weeks prior to presentation, a painful new lump formed within her right axilla. During her intake history and physical, a large right breast mass was appreciated. The warm and tender right breast exhibited an erythematous, peau d'orange appearance. Significant axillary adenopathy was also present. Additionally, the patient had a family history of breast cancer in her maternal cousin and maternal aunt, both of which were diagnosed under the age of 50. At initial presentation, computed tomography (CT) of the thorax revealed a hyperattenuating mass within the outer portion of the right breast tissue that measured approximately 2.3 cm with axillary, pectoral, and clavicular primary and metastatic lymphadenopathy. A primary breast malignancy was suspected and follow-up breast consultation and biopsy were recommended.

**Figure 1:**
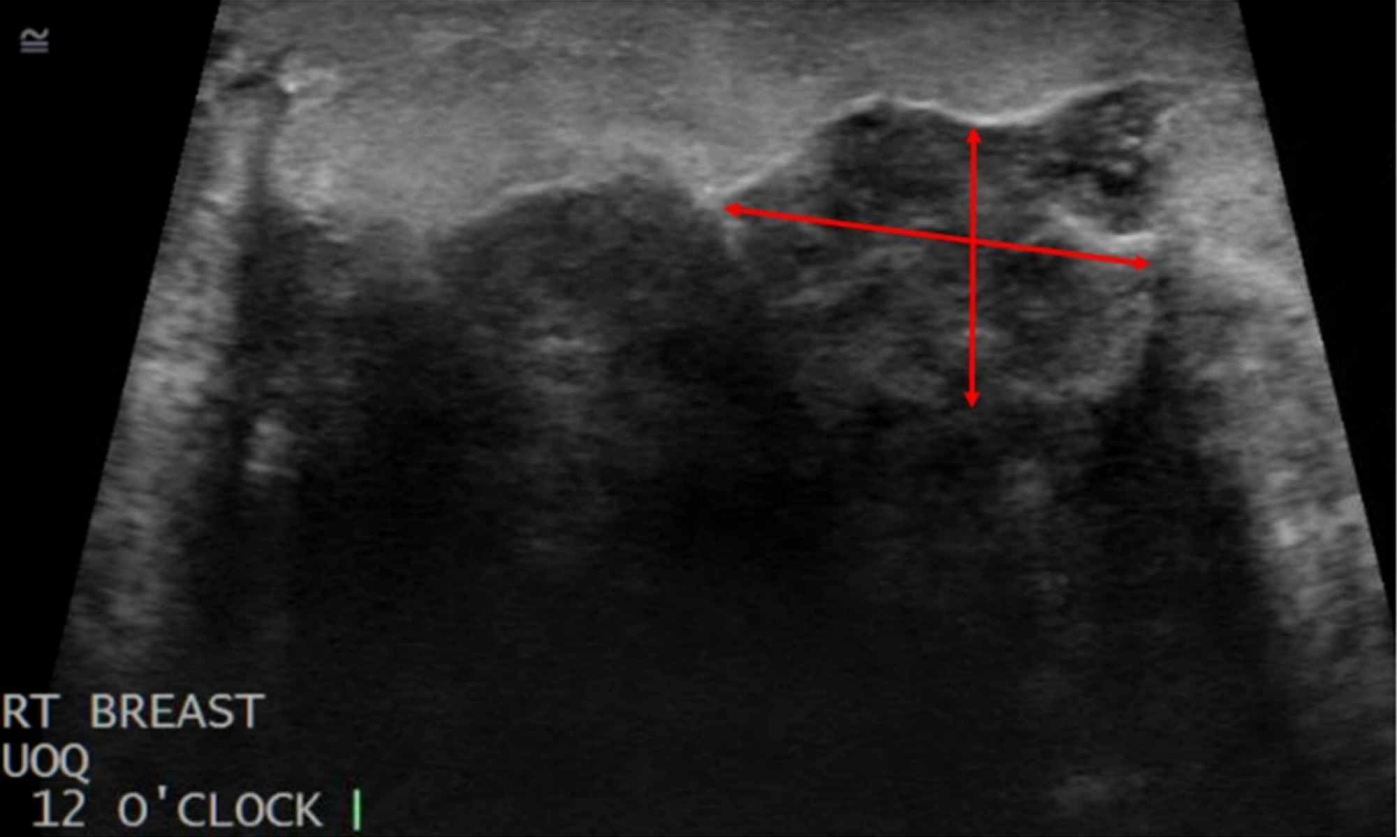
Diagnostic Right Breast Ultrasound An ultrasound of the right breast revealed an expansive mass involving all four quadrants of the breast. A portion of the mass located in the upper outer quadrant (UOQ) is imaged here, with the diameter of the mass being defined by the red arrows.

**Figure 2:**
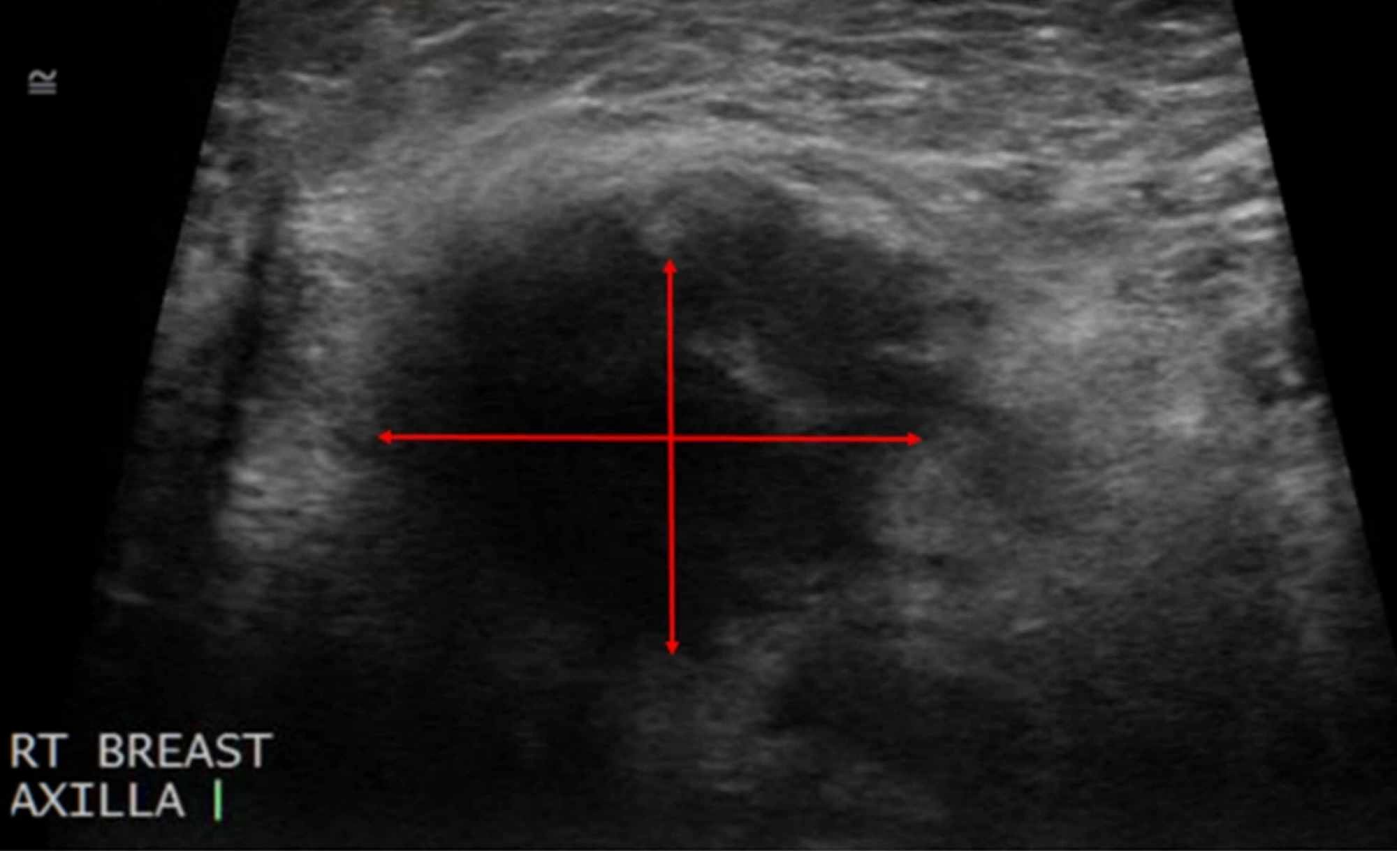
Ultrasound of the Right Axillary Region A hypoechoic mass, measuring 3 centimeters, is present in the axilla with the diameter represented by the red arrows.

Unfortunately, the patient did not proceed with the recommended diagnostic plan due to financial circumstances and presented a year later to our clinic. At this time, the patient was at nine weeks gestation and was being evaluated for her initial prenatal visit. During this visit, the patient endorsed concern regarding the right breast mass, which had now doubled in size in the past six months. She reported significant pain, burning, and shortness of breath. A targeted ultrasound of the right breast and axillary region were performed and revealed an expansive mass involving all four quadrants of the breast, extending from posterior depth to the nipple with suspected nipple invasion (Figures 1-2).

**Figure 3:**
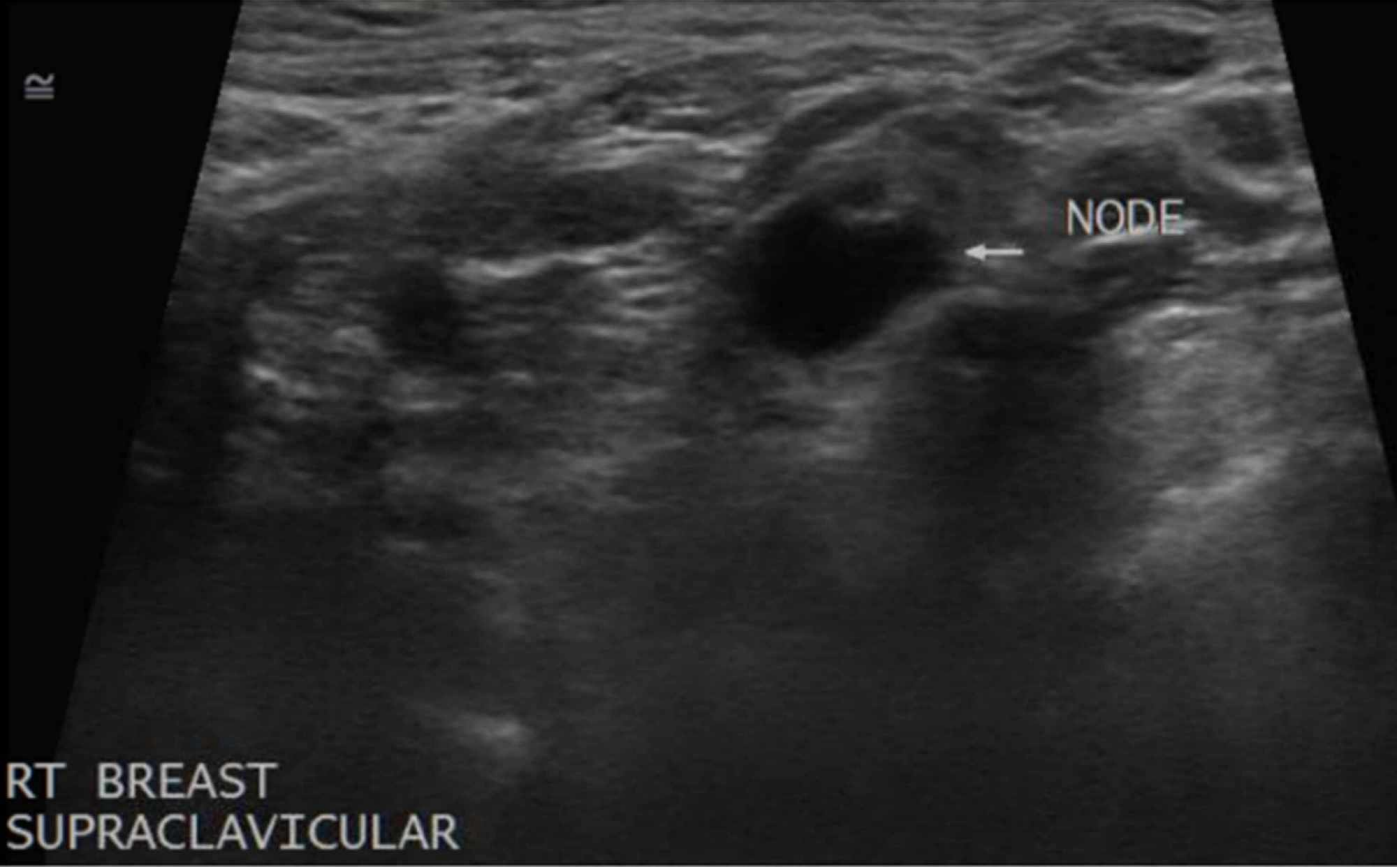
Supraclavicular Lymphadenopathy An ultrasound of the right supraclavicular region reveled several enlarged lymph nodes, one of which is imaged here (white arrow).

**Figure 4:**
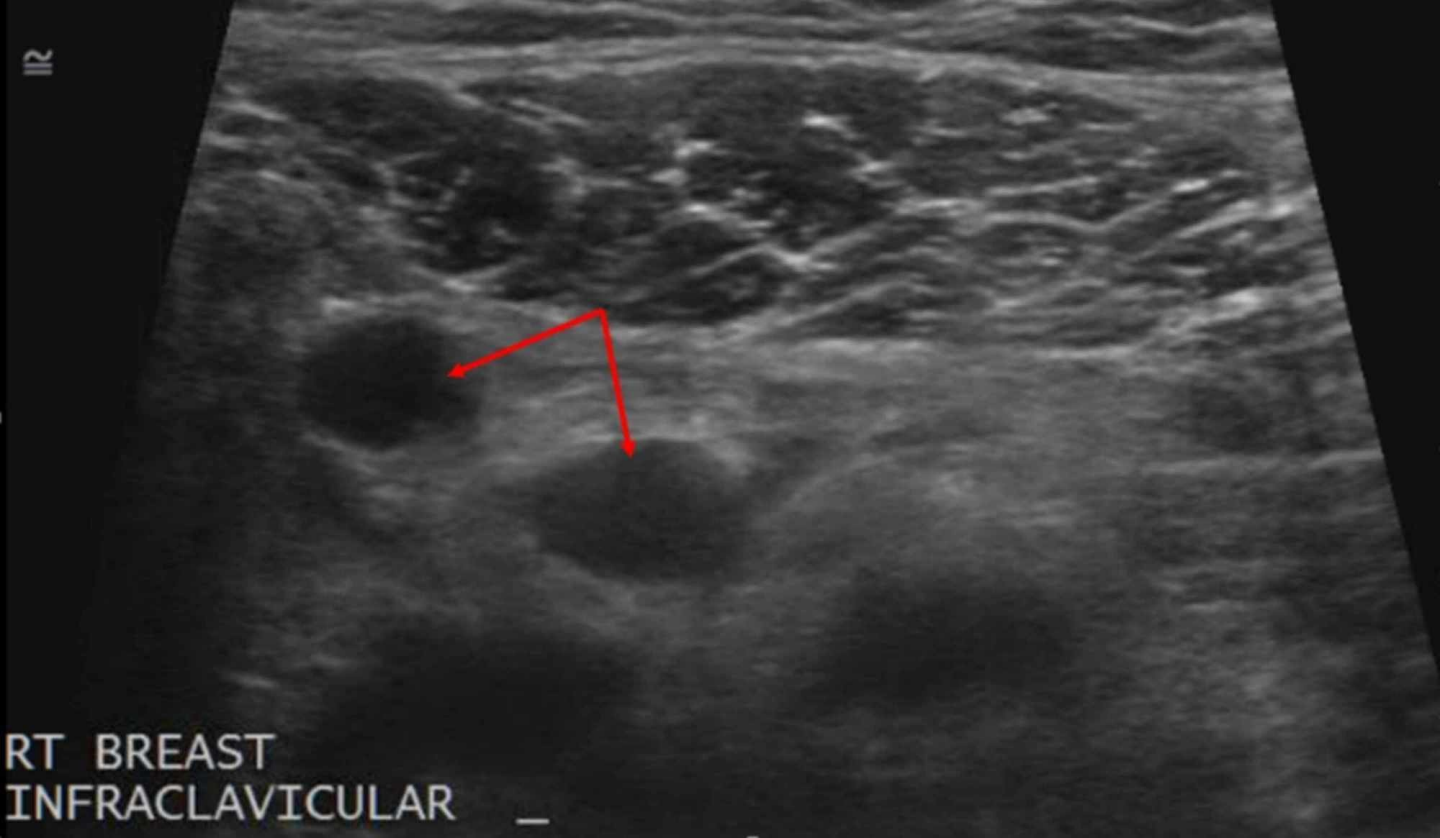
Infraclavicular Lymphadenopathy An ultrasound of the right infraclavicular region revealed multiple enlarged lymph nodes, two of which are imaged here (red arrows).

The axillary mass and multiple abnormal supraclavicular and infraclavicular lymph nodes were highly suspicious for metastatic disease (Figures 3-4).

**Figure 5:**
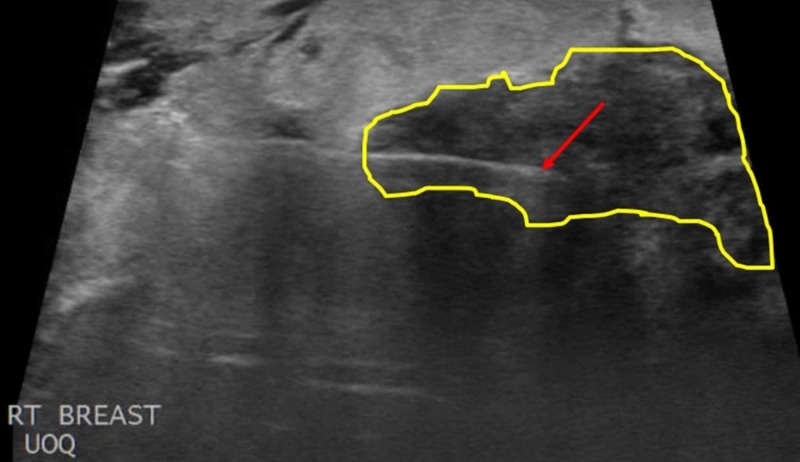
Ultrasound-Guided Core Biopsy of the Right Breast Mass An ultrasound-guided core biopsy was performed of the right breast mass. The needle tip, designated by the red arrow, was inserted into the upper outer quadrant (UOQ) of the right breast and a biopsy was taken of the mass (yellow outline).

**Figure 6:**
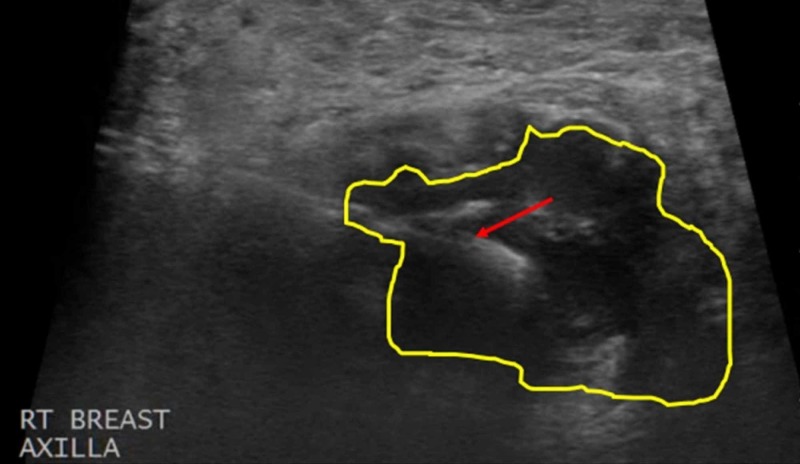
Ultrasound-Guided Core Biopsy of the Right Axillary Mass A core biopsy of the right axillary mass was performed using ultrasound guidance. The needle tip, designated by the red arrow, was inserted into the axillary region and a biopsy was taken of the mass (yellow outline).

The breast mass was given Breast Imaging-Reporting and Data System (BI-RADS) category five, highly suggestive of malignancy, and the patient subsequently underwent core biopsy of the conglomerate of masses (Figures 5-6).

The morphological findings of the biopsy sections showed poorly differentiated invasive ductal carcinoma with a Bloom-Richarson grade three score within the right breast and invasive lobular carcinoma.

The patient was given an appointment with medical oncology, as well as maternal-fetal medicine for the management of a high-risk pregnancy. Following a group discussion with insight from surgical oncology, breast imaging, medical oncology, radiation oncology, surgical pathology, and maternal-fetal medicine, a consensus was made for treatment recommendations. However, treatment options were limited as she was not a suitable candidate for the standard of care treatment for invasive ductal carcinoma of the breast due to her gestational status and insufficient financial funding.

## Discussion

The diagnosis and treatment of breast cancer during pregnancy presents a challenging situation for patients, their families, and caregivers. The initial diagnostic process can be complicated by the physiological changes that occur in the breast during pregnancy. Pregnancy can cause multiple changes within the breast, including increased glandularity, size, and density of the breast tissue [3]. Although pregnant women may present with similar physical examination findings as compared to nonpregnant breast cancer patients, a delay in cancer diagnosis in pregnant breast cancer patients is often secondary to pregnancy-related changes of breast tissue. Thus, pregnant breast cancer patients often present with an advanced disease stage and axillary lymph node involvement. Given the concern for delayed diagnosis, palpable masses persisting over two weeks during pregnancy should be investigated, although it is reported that approximately 80% of breast biopsies during pregnancy will be benign [4].

Mammography has been successfully used during pregnancy when done with proper fetal shielding. However, the increased density of the breast during pregnancy may lower the sensitivity of mammography [3]. Therefore, ultrasonography is an attractive choice for the diagnosis of breast cancer during pregnancy. There isn't any radiation exposure risk for the fetus, and ultrasonography is not limited to dense breasts, which could obscure masses on mammography. Breast magnetic resonance imaging (MR) has not yet been prospectively studied for the diagnosis of breast masses in pregnant or lactating women due to concerns over gadolinium exposure in pregnant patients.

Any clinically suspicious mass should be biopsied even if the ultrasound and mammogram are nondiagnostic. Core biopsies can be performed safely under local anesthesia. Unfortunately, the patient, in this case, presented with advanced malignancy of the breast prior to becoming pregnant but was unable to obtain the standard medical diagnosis and treatment due to her financial situation. Eventually, when she returned a year later at nine weeks gestation, she received a diagnostic ultrasound and core biopsy of the right breast.

At this time, the patient was seen by medical oncology who recommend pregnancy termination, given her advanced cancer and positive human epidermal growth factor receptor two (HER2) status. Nonpregnant patients with HER2 positive tumors may receive targeted therapy with trastuzumab; however, this form of therapy is highly contraindicated in pregnancy. Zagouri et al. presented a systematic review and meta-analysis aimed to evaluate the safety of trastuzumab during pregnancy. In the 17 studies included, the occurrence of oligohydramnios or anhydramnios was the most common (61.1%) adverse event [5]. Seventy-three point three percent (73.3%) of pregnancies exposed to trastuzumab during the second or third trimester were complicated with oligohydramnios or anhydramnios [5]. Only in 52.6% of cases was a healthy neonate born [5]. Therefore, trastuzumab should not be administered during pregnancy and is not a standard treatment in this case [5].

If the patient declines pregnancy termination, a surgical approach in management is indicated. Current guidelines recommend mastectomy with axillary lymph node dissection over lumpectomy and radiation, as radiation is contraindicated in pregnancy. However, the patient, in this case, presented with non-operable metastatic disease, rendering surgical management an undesirable option.

If the patient were to continue the pregnancy, medical oncology recommended chemotherapy at the beginning of the second trimester. The decision to use chemotherapy in a pregnant breast cancer patient should depend upon the patient's disease stage and tumor characteristics. Most chemotherapy agents are rated pregnancy category D, suggestive for positive evidence of human fetal risk. However, in life-threatening situations, or in sight of serious disease, category D drugs may be acceptable for use despite its risk. Because reports of fetal malformations have been in the range of 14%-19% when chemotherapy has been given in the first trimester, it is highly recommended to administer chemotherapy in the second and third trimesters [6]. The reported frequency for fetal malformation during the second and third trimesters decreases to 1.3% [6]. This is likely due to the fact that organogenesis is complete by the second trimester, decreasing the risk of fetal malformation during this time of gestation.

Of the chemotherapeutic options, the use of anthracyclines during pregnancy has been the most widely studied and accepted form of treatment for advanced breast cancer during pregnancy. The MD Anderson Cancer Center has been prospectively treating women diagnosed with breast cancer during pregnancy with a standardized protocol, called the Fluorouracil, Adriamycin and Cytoxan regimen, since 1989 [7-8]. It is further recommended that chemotherapy be terminated after the 35th week of gestation, to decrease the risk of developing sepsis and hemorrhage, due to myelosuppression at the time of delivery. Within this study, 57 pregnant women were treated for breast cancer using this protocol and all women had live births. Of the children, three had reported congenital malformations [7]. 

In the presented case, the recommended chemotherapy regimen included the use of doxorubicin and cyclophosphamide. The patient denied both the abortive approach and chemotherapy option. With limited options and an inability to secure funding, early delivery at 32 weeks was suggested. This would allow the early initiation of chemotherapy treatment postpartum while still optimizing both fetal and maternal outcomes. Because the pregnancy was not threatening the patient's life, there was no indication for early delivery prior to 32 weeks. Manuck et al. presented a large study reporting that the frequency of all morbidities fell beyond 32 weeks gestation [9]. Neonatal length of hospital stay decreased significantly with each additional completed week of pregnancy; among babies delivered from 26 to 32 weeks of gestation, each additional week in utero reduced the subsequent length of neonatal hospitalization by a minimum of eight days [9]. Moreover, for infants born at 32 weeks, 0.2% died, 8.7% had major morbidity, 76% had minor morbidity, and 14.9% survived with no morbidity [9]. Balancing the risk of prematurity and the risk of continuing a pregnancy, in this case, is a difficult decision to make. Given the patient’s medical and financial condition, it was not recommended to deliver the infant prior to 32 weeks. Some studies in the literature even recommend against delivery prior to 36 weeks gestation in the absence of an obstetric indication.

Although the patient was given medical advice and counseling consistent with current guidelines, she was not willing to accept these recommendations and was lost to follow-up. Overall, the presented patient demonstrates the challenge of diagnosing and treating metastatic breast cancer in pregnant women, further complicated by financial burdens.

## Conclusions

The diagnosis of breast cancer in pregnancy is an uncommon but devastating diagnosis that is likely to increase in incidence. Data regarding acceptable protocols and guidelines for the treatment of breast cancer during pregnancy are becoming more readily available. As the medical community continues to add to this growing body of literature, healthcare providers are able to offer the best care possible for these patients and their families.
